# Natural Light Exposure as a Potential 7th Pillar of Lifestyle Medicine

**DOI:** 10.1177/15598276261485285

**Published:** 2026-09-08

**Authors:** Marie Tremblay-Laliberté, Angelo Tremblay

**Affiliations:** 1 5622 Faculty of Environmental Planning, School of Design, Université de Montréal, Montreal, QC, Canada (MTL); 2 4440 Faculty of Medecine, Department of Kinesiology, Université Laval, Quebec City, QC, Canada (AT)

**Keywords:** biological clock, circadian, clinical practices, illumination, melatonin, sleep

## Abstract

A growing number of studies highlight the observed effects of natural light exposure on health and bodily functioning, as well as its added value for the performance of daily activities. Although natural light exposure is gaining increasing recognition as a determinant of health and well-being, it remains underrepresented in the field of lifestyle medicine and should be considered as a potential new pillar alongside a healthy diet, physical activity, restorative sleep, stress management, positive social relationships, and the avoidance of risky substances. This narrative review aims to synthesize the main findings of the literature and relevant conceptual frameworks that support the inclusion of daily exposure to natural light as a component of lifestyle medicine and its integration into clinical practice. Further research is required to better characterize the effects of natural light exposure on physical and mental health and the overall evidence presented in this article supports continued investigation in this field.

“Current evidence consistently indicates that regular exposure to natural daylight constitutes the most effective means of meeting intrinsic human light requirements and of supporting overall health and well-being.”

## Introduction

Natural light, an essential and often underestimated environmental factor, exerts pervasive and largely non-conscious effects on human physiology and is fundamental to health and life itself.^1^ The human organism is biologically calibrated to the natural light cycle, and a growing body of research highlights the effects of natural light exposure on human health and well-being. Across the 24-hour light–dark cycle, natural light contributes to the regulation of multiple physiological functions, including core body temperature, hormonal secretion, and sleep–wake regulation.^2^ Evidence also indicates that natural light influences brain function and behavior, affecting parameters such as work performance, mood, and stress levels.^3^ Due to its effects on multiple physiological systems, exposure to natural light is associated with the prevention of several diseases and is increasingly incorporated into treatment strategies for mental health disorders such as anxiety and depression.^4^ Daily exposure to natural light therefore represents a key determinant of health and well-being, closely linked to lifestyle behaviors and daily routines. In this context, it may be considered as a potential seventh pillar of lifestyle medicine, alongside healthy nutrition, physical activity, restorative sleep, stress management, positive social relationships, and avoidance of risky substances. While nature exposure has recently been proposed as a potential additional pillar of lifestyle medicine,^5^ natural light exposure remains underrepresented in this field and warrants further investigation regarding its health benefits and its impact on daily activities. This narrative review aims to synthesize key findings from the literature and relevant conceptual frameworks supporting the inclusion of daily natural light exposure as a component of lifestyle medicine and its integration into clinical practice.

### The Circadian System and Non-Visual Light Perception

Periodic oscillations are characteristic of biological processes, many of which are synchronized with the light–dark cycle, commonly referred to as circadian rhythms.^6^ In this context, light acts as a primary zeitgeber, enabling the proper alignment of the circadian system with the day–night cycle. Accordingly, human physiology exhibits circadian patterns that are directly influenced by variations in ambient light throughout the day. A tangible link supporting the role of daylight in health has been provided by the discovery of intrinsically photosensitive retinal ganglion cells (ipRGCs), which project directly to the suprachiasmatic nucleus (SCN), the site of the central biological clock.^3^ This non-image-forming photoreceptive system is distinct from the visual system, and its localization within the inner retina suggests that it does not contribute to image formation but instead plays a key role in non-visual photoreceptive functions, including hormone regulation, alertness, cognitive performance, and affective responses.^7^ It has also been suggested that light exposure influences central serotonergic turnover via retinal stimulation.^8^ Beyond circadian entrainment, light exposure affects cardiovascular function, core body temperature,^9,10^ and has been associated with sleep quality.^11,12^ Moreover, the circadian system, beyond regulating sleep, exerts autonomous control over nearly all physiological systems, modulating organ function across the sleep–wake cycle. Light exposure is therefore essential for the temporal organization of bodily functions and for supporting physiological recovery processes. The discovery of non-visual effects of light on the brain and multiple physiological systems has strengthened the links between light exposure and health. Current evidence suggests that maximizing daytime light exposure, minimizing nocturnal light exposure, and maintaining regular light–dark cycles that support circadian alignment may contribute to optimal health outcomes.

### Variability in the Effects of Light Exposure

Light exposure and its potential health effects are highly dependent on the exposure context. Significant differences exist between natural (sunlight) and artificial light exposure, as well as between indoor and outdoor environments. Geographically, photoperiod (daylight duration) varies with latitude and season.^13^ The timing of light exposure is also a critical determinant of its physiological effects. Morning sunlight exposure has distinct effects compared with late-day exposure on sleep–wake regulation and circadian alignment.^14^ In addition to timing, duration, intensity, wavelength, and prior light exposure history all act as modulatory factors that influence individual responses to light. Daytime light exposure history is particularly important, as it shapes evening light sensitivity and contributes to either the maintenance or disruption of the sleep–wake system. Evidence from studies on photic history in human circadian regulation suggests that daytime light exposure can enhance nocturnal melatonin secretion, strengthen the biological clock, and reduce sensitivity to evening and nighttime light exposure.^15,16^ Furthermore, individual factors such as age, sex, and chronotype have also been shown to modulate responses to light. For example, a 75-year-old individual requires approximately three times more light than a 45-year-old to elicit the same circadian response.^17^ Sex-related differences have been observed in certain responses to light, including melatonin suppression, alertness, and sleep. Women’s stronger response to bright light (400 to 2000 lux) suggests that their circadian system may be more sensitive than men’s to the effects of daylight on this system.^18^ Overall, the context-dependent and highly variable nature of light exposure effects makes it difficult to define universal thresholds for “healthy” light exposure. Nevertheless, a substantial body of research has focused on identifying the beneficial effects of natural light exposure on health and on elucidating the factors influencing circadian rhythm regulation. The following table summarizes the general conditions influencing the effects of light on the circadian system.

Table of common condition affected by light.

**Table:** 

Category	Condition
Light characteristics	Intensity, wavelength, exposure duration
Timing	Timing of exposure, season/photoperiod, exposure history
Individual characteristics	Chronotype, age, sex
Reception characteristics	Viewing angle/direction of light

### Health Benefits of Sun Exposure

Sun exposure is associated with a wide range of beneficial and protective effects on health. Widely used in the early 20th century to treat conditions such as tuberculosis and rickets, heliotherapy, or sun therapy, is still employed today to support the management of certain diseases.^19^ The beneficial effects of natural light exposure are frequently attributed to vitamin D production, often referred to as the “sunshine hormone.” Vitamin D is an essential component of cellular biological processes, contributing to the regulation and optimal functioning of physiological systems. In particular, it plays a key role in nutrient absorption and in maintaining calcium and phosphorus levels, which are critical for bone health. Vitamin D status is strongly influenced by sun exposure. Insufficient exposure leads to reduced vitamin D levels and impaired cellular responses to both internal and external signals,^20^ which may contribute to a range of health outcomes, including cancer, cardiovascular disease, influenza, diabetes, and certain autoimmune disorders.^21^ Recent studies have advanced understanding of how UV radiation induces the release of cutaneous mediators that alter the skin and blood transcriptome, including immune-related genes.^22^ These findings suggest that the beneficial effects of sunlight are likely mediated by multiple interacting pathways rather than vitamin D alone. Moreover, sun exposure may involve additional physiological mechanisms that remain insufficiently explored, including nitric oxide release, beta-endorphin production, and circadian rhythm regulation.^23^ The following section presents examples of studies linking solar exposure to beneficial health outcomes. In most cases, serum vitamin D concentrations are used as an indicator for sun exposure, allowing for the identification of associations between sun exposure and disease prevention.

#### Life Expectancy and Mortality

A study conducted by Lindqvist, Epstein et al. (2014)^24^ found that women with higher sun exposure had a twofold lower mortality rate and a life expectancy 2 to 3 years longer than those who avoided sun exposure. Moreover, the study showed that non-smoking women who avoided sun exposure had a life expectancy similar to that of smokers in the group with the highest sun exposure. This finding suggests that avoiding sun exposure is a risk factor for mortality of a magnitude comparable to that associated with tobacco use. There is now compelling evidence linking vitamin D levels above 40 ng/mL (100 nmol/L) to a reduced risk of many types of cancer and several other diseases, as well as to increased life expectancy. In addition, mortality rates increase by approximately 25% in winter compared to summer in the United States.^25^

#### Cardiovascular Diseases

Emerging evidence suggests an association between sun exposure, blood pressure, and cardiovascular disease.^26^ Low sun exposure is associated with a twofold increase in cardiovascular mortality risk compared with higher exposure levels,^24^ while the incidence of daytime myocardial infarction appears to decline with increasing summer solar radiation.^27^ Epidemiological studies have also reported latitude- and season-dependent variations in the incidence and severity of several cancers and cardiovascular diseases.^28,29^

#### Metabolism

Geldenhuys et al. (2014)^30^ suggest that exposure to ultraviolet (UV) radiation may represent an effective means of attenuating obesity and metabolic syndrome through mechanisms independent of vitamin D, but dependent on other UV-induced mediators such as nitric oxide. This study highlights the capacity of UV radiation to significantly reduce weight gain, in contrast to vitamin D supplementation. These findings suggest that low vitamin D status in obese individuals may primarily reflect reduced UV exposure, providing new insight into earlier studies reporting a consistent inverse association between body mass index and serum 25(OH)D levels.^31^ Afzal et al. (2013)^32^ reported that low serum 25(OH)D concentrations are associated with a 35% increased risk of type 2 diabetes. This observation is consistent with our findings, which demonstrated an inverse relationship between circulating 25(OH)D levels and various markers of adiposity.^33^

#### Attention Deficit/Hyperactivity Disorder (ADHD)

A study by Arns et al. (2013)^34^ established a formal association between ADHD prevalence and solar irradiance, showing higher prevalence in regions with lower sunlight exposure. After adjustment for known ADHD-related confounders, regions with higher solar irradiance (including the United States and other countries) exhibited lower ADHD prevalence, suggesting that sun exposure may exert a protective effect against the disorder. These findings further support a potential link between the preventive effects of solar irradiance and improvements in disorders associated with circadian rhythm disruption, which have recently been implicated in ADHD.

#### Alzheimer’s Disease and Related Dementias (ADRD)

Clinical studies have demonstrated that exposure to bright light during the day can help stabilize sleep and activity rhythms in individuals with dementia-related disorders. Morning exposure to bright light (typically >1000 lux at the cornea) has been shown to improve nocturnal sleep, enhance daytime alertness, reduce evening agitation, and strengthen rest–activity rhythms in patients with Alzheimer’s disease and related dementias.^35^ Keeney et al (2013)^36^ reported that vitamin D deficiency may contribute to cognitive decline in middle-aged and older adults. In a prospective cohort study of 1658 Americans aged 65 years old followed over a period of 6 years, Littlejohns et al. (2014)^37^ found that individuals with very low vitamin D levels (<10 ng/mL) had more than twice the risk of developing Alzheimer’s disease compared with those with higher levels (>20 ng/mL), with similar findings observed for overall dementia risk. The authors highlighted that this was among the first large-scale studies to examine the association between Alzheimer’s disease and circulating vitamin D concentrations. These findings are consistent with previous evidence linking low vitamin D status to cognitive decline.^38^

#### Seasonal Depression

An increasing amount of research suggests that insufficient light exposure may contribute to the development of seasonal affective disorder (SAD).^39^ This condition, which is more prevalent in regions with reduced seasonal sunlight, is associated with symptoms such as low mood, irritability, anxiety, lethargy, metabolic dysregulation, and hypersomnia. Among established treatments for SAD is timed exposure to bright light.^40^ Light therapy is currently most commonly delivered using high-intensity artificial light designed to approximate natural daylight. However, Wirz-Justice et al. (1996)^41^ showed that, depending on latitude, winter daylight itself may be effective in reducing SAD symptoms, even under overcast conditions. Mental health benefits have also been reported in indoor light exposure contexts. For instance, exposure to indirect daylight through windows has been associated with reduced depressive mood and increased workplace productivity.^42^ Nevertheless, despite greater exposure-related risks compared with indoor settings, outdoor natural light exposure appears more effective in supporting vitamin D synthesis, circadian realignment, and has been associated with reduced stress and anxiety levels.^3^

#### Additional Health Benefits of Sun Exposure

In addition to the beneficial effects described above, research has associated sun exposure with a reduced risk of developing several diseases and health conditions, including breast cancer,^43^ food allergies,^44^ dental caries,^45^ SARS-CoV-2 infection,^46^ and multiple sclerosis.^47^ Furthermore, sun exposure has been shown to increase serotonin levels^48^ and to regulate melatonin production^49^ with additional reported benefits for mental health and mood regulation.^42^

### Risks of Sun Exposure

Excessive and inappropriate sun exposure may have adverse health effects by increasing the risk of skin cancers, particularly malignant melanoma.^4^ Sunburns have also been consistently associated with a higher risk of melanoma. To minimize the harmful effects of excessive sun exposure, protective measures are recommended, including avoiding sunburn and limiting unprotected exposure beyond approximately 30 minutes, although optimal exposure duration varies according to latitude and skin phototype. In this context, intermittent unprotected sun exposure of approximately 15 min, 2-3 times per week between 11:00 and 15:00, may be sufficient for individuals with skin type II (clear skin) living at northern latitudes to meet annual vitamin D requirements.^50^ In a review of the risks and benefits of sun exposure, Hoel, Berwick et al. (2016)^23^ highlighted the need to move beyond recommendations focused solely on sun avoidance toward a more balanced perspective recognizing that non-burning sun exposure may confer health benefits. Evidence further indicates that the ultraviolet radiation doses required for vitamin D synthesis are substantially lower than those required to induce sunburn and that an adequate use of sunscreens prevent sunburn yet allowing vitamin D synthesis.^51^ During periods of high UV radiation, gradual exposure is recommended, ranging from approximately 5 to 30 min per day depending on skin type and UV index, along with appropriate protective clothing and the use of sunglasses that filter UV radiation to protect the eyes.^52^ Adverse effects of sun exposure may also be exacerbated under certain conditions, such as during the use of medications or in individuals diagnosed with photosensitivity disorders or sun-related allergies or intolerances.

### Light exposure and contemporary lifestyle patterns

Lifestyle and environmental factors play a significant role in the relationship between light exposure and health. Today, reduced direct contact with nature has led individuals to spend approximately 90% of their time indoors under low-intensity artificial lighting. Although this level of illumination is generally sufficient for visual tasks, it does not produce sufficient stimulus for the circadian system entrainment as artificial lighting typically ranges between 200 and 500 lux, whereas natural sunlight can reach approximately 100,000 lux in full sunlight conditions. While the circadian system was originally synchronized by sunlight, contemporary lifestyle changes and artificial lighting have altered daily light exposure patterns, shifting and disrupting circadian regulation. In this context, light exposure can have adverse effects when it occurs at biologically inappropriate times, leading to circadian misalignment. These effects are mainly associated with artificial light, as daylight is typically available at biologically appropriate times.^3^ This response to altered light exposure is referred to as “circadian disruption” and has been associated with a range of negative health outcomes beyond sleep disturbances.^53^ The following table presents illuminance levels under various natural lighting conditions, as well as illuminance levels associated with artificial lighting across different applications. The comparison highlights the substantially higher illuminance levels that can be achieved through daylight exposure compared with those typically provided by functional artificial lighting.

Table of common lux level (natural and artificial light).

**Table:** 

Location/Condition	Typical Illuminance (lux)	​	​
Overcast outdoor day	1000-5000	Classroom and office work space	300-500
Sunrise/sunset	400-4000	Retail store	300-750
Early morning or late afternoon (clear)	2000-10,000	Kitchen countertop	300-750
Full daylight (window indirect sun)	10,000-25,000	Hospital examination room	500-1000
Direct sunlight	32,000-100,000	Parking garage	50-100
Urban street (clear day)	10,000-50,000	Laboratory	500-1000
City park	20,000-80,000	Residential hallway	30-100

#### Artificial Light at Night (ALAN) and Adverse Health Effects

Several studies have examined the effects of nighttime light exposure on health and circadian rhythm functioning. In most cases, melatonin concentrations are used as an indicator for light exposure, allowing for the identification of associations between ALAN and various health outcomes. The most commonly documented effects of artificial light at night (ALAN) on health include circadian disruption and suppression of nocturnal melatonin production, a key hormone involved in sleep regulation.^54^ ALAN exposure has also been associated with an increased risk of several diseases, including cancer,^55–58^ as well as cardiovascular disease, diabetes, mood disorders, and obesity.^59–62^ More recently, a Harvard research group reported that circadian disruption induced by artificial light at night was associated with a 30-50% increased risk of myocardial infarction, stroke, and heart failure.^63^ These findings suggest that ALAN may represent an emerging cardiovascular risk factor to be considered alongside established lifestyle determinants such as smoking, alcohol consumption, diet, physical activity, and sleep. Overall, this study indicates that exposure to artificial light at night may independently predict cardiovascular disease risk beyond traditional risk factors, highlighting ALAN as a potentially important dimension of modern lifestyle requiring further consideration in preventive health strategies. In a 2023 literature review, Figueiro and Pedler^64^ highlight the relationship between exposure to the natural light–dark patterns and its potential role in the prevention of cardiovascular disease. The authors emphasize the adverse effects of insufficient exposure to natural light associated with modern lifestyles, as well as the health consequences of circadian misalignment which disrupting sleep patterns and other physiological processes, may contribute to the increased prevalence of cardiovascular disease. Exposure to an appropriate pattern of light and darkness is essential for the effective regulation of the body’s circadian system that requires substantial exposure to bright light during the day, limited exposure to light in the evening, and complete darkness during the night in order to function optimally. Accordingly, the authors identify specific lifestyle behaviors that can promote circadian synchronization, enhance sleep quality, and potentially reduce the risk of adverse health outcomes, including cardiovascular disorders. These practices include maintaining a consistent sleep–wake schedule, seeking exposure to morning light, increasing exposure to natural light throughout the daytime (even through a window), and limiting exposure to artificial light during the evening. Collectively, these measures may support the proper alignment of the circadian system and contribute to improved cardiovascular and overall health.

Excessive evening use of screens and smartphones has been associated with sleep disturbances, partly due to blue light–induced suppression of melatonin secretion.^65^ The circadian system is particularly sensitive during the evening and night, when it exhibits a heightened response to short-wavelength (blue) light. It is of interest to relate these observations to the study by Rangtell et al. (2016),^66^ in which 14 participants were instructed to read in the evening using an electronic tablet for 1 week, and then a printed book for another week, following prior daytime exposure to bright light (6.5 hours at approximately 569 lux). No significant differences were observed between the two reading conditions in sleep parameters or salivary melatonin levels prior to bedtime. These results suggest that daytime bright light exposure may mitigate the potential adverse effects of evening blue light exposure on sleep. This pattern reflects circadian system adaptation to variations in light intensity and spectral composition over longer timescales than the visual system. Current responses to light are influenced by prior light exposure history, and these effects may persist for several hours or even days.^67^

Shift work, which affects nearly one in five workers in industrialized countries, leads to circadian misalignment associated with a broad range of adverse health outcomes, from cognitive impairment to cancer, as well as reduced quality of life.^68^ A study conducted at McGill University involving 60 nurses found that exposure to bright light before night shifts reduced fatigue and its impact on work performance. In addition, evening bright light exposure was associated with improved post-shift sleep quality among night-shift nurses.^69^

#### Light Exposure Conditions in Interior Environments and Health Outcomes

In recent years, an expanding body of knowledge has examined light exposure conditions in living environments and their effects on occupants’ health and well-being. A literature review by Urrutia-Moldes (2025)^70^ on the non-visual effects of light in correctional environments highlighted the negative consequences of limited daylight exposure and excessive reliance on artificial lighting (with a blue-enriched spectral composition) on circadian regulation, sleep quality, mood, and prisoners’ mental health. Accordingly, neglecting lighting design in correctional facilities may contribute to adverse psychological outcomes. Conversely, the study suggests that architectural and environmental strategies that optimize daylight exposure and incorporate adjustable artificial lighting with appropriate spectral characteristics may promote emotional stability, reduce aggression, and support rehabilitation outcomes.

A study by Boubekri et al. (2014)^71^ examined the relationship between workplace light exposure conditions and employees’ sleep quality, physical activity patterns, and overall quality of life among office workers. The findings indicated that individuals working in proximity to a window were not only exposed to greater levels of light during working hours but also obtained approximately 46 minutes more sleep per night compared to those in windowless environments. The results of this study suggest that exposure to natural light within the workplace may have implications extending beyond the immediate work setting, particularly by influencing both sleep duration and sleep quality. Accordingly, the study raises the possibility that access to—or deprivation of—natural light in office environments may have broader consequences for additional health-related outcomes.

A study by Walch et al (2005)^72^ examined the effects of exposure to natural light on the overall recovery process and analgesic medication use among patients undergoing spinal surgery. The study found that patients hospitalized in rooms with greater exposure to natural light consistently required less analgesic medication, regardless of age. These findings underscore the importance of incorporating natural light into the design of the built environment, particularly in healthcare settings, to support patient recovery, health, and well-being.

A 2023 literature review by Lech et al.^73^ examines how light exposure influences students’ health and well-being, as well as their core academic outcomes, through a range of mechanisms, including the regulation of circadian rhythms. This study highlights that current lighting conditions experienced by students are suboptimal from the perspective of biological mechanisms and the non-visual effects of light, and that these conditions could be improved through interventions aimed at achieving better biological alignment between individuals and their learning environments. The potential interventions include the incorporation of outdoor instructional activities, as well as the integration of architectural features—such as skylights, glazing that permits greater transmission of ultraviolet light, and larger windows—into classroom design to increase students’ exposure to natural light. It is also suggested to use artificial lighting that emits a broader spectrum of light, better approximating the wide spectrum of natural sunlight. These measures, while relatively accessible in terms of required investment and planning, could yield substantial benefits for students, both in improving health and well-being and in enhancing academic outcomes. By linking student health and well-being, learning activity, and the design of educational lighting environments, this review recommends that educational institutions incorporate light exposure conditions into the planning and design of their educational programs and their architectural environments.

These studies highlight the importance of applying current knowledge on light exposure and health into planning and design practices—including architecture, interior design, and urban planning—to improving quality of life and to advancing preventive health strategies. Recent advances in artificial lighting technologies have enabled the partial mitigation of light exposure deficiencies in indoor environments and have been associated with improvements in certain aspects of human health. Nevertheless, natural light cannot be fully replicated or substituted, and claims to the contrary remain unsupported. Current evidence consistently indicates that regular exposure to natural daylight constitutes the most effective means of meeting intrinsic human light requirements and of supporting overall health and well-being.

#### Natural Light Exposure and Daily Activities

The inclusion of natural light exposure as a component of lifestyle medicine is accompanied by the challenge of engaging in activities that broadly promote healthy living. Outdoor physical activity practice offers numerous possibilities of natural light exposure. Relevant examples include hiking, walking and jogging, biking, cross country skiing, and team sports such as soccer. Recently, we have focused on one of these activities by evaluating the lifestyle and health-related profile of individuals who regularly practice gardening. Analysis of their lifestyle habits revealed several characteristics that, beyond light exposure, may contribute to overall health benefits, including regular physical activity, frequent consumption of fruits and vegetables, and healthy sleep habits. Participants also reported good emotional well-being and high levels of satisfaction with gardening and contact with natural environments. It is noteworthy that the prevalence of obesity and diabetes in this group was substantially lower than that reported in the Canadian population (Tremblay et al., unpublished data). Although these findings do not establish causality regarding the health benefits of gardening, they add to the growing body of evidence suggesting that outdoor activities involving natural light exposure may contribute to improved well-being and health outcomes.

A study conducted by Zebowska et al. (2024)^74^ investigated the associations between the timing and duration of morning dog walking and the risk of depression among nurses, while also examining effect modification according to chronotype (morning vs evening types). The authors hypothesized that dog ownership and engaging in morning dog walking would be associated with a lower risk of depression, particularly among individuals with an evening chronotype, by facilitating better alignment of their circadian rhythms with the solar cycle. The findings further suggest that individuals with an evening chronotype may derive greater mental health benefits from morning dog walking compared to those with a morning chronotype. Overall, dog ownership alone was not associated with depression risk, although a higher risk was observed among evening chronotypes. Nevertheless, this study indicates that morning dog walking may help reduce depression risk in evening-type individuals, while emphasizing that additional research is needed to confirm these findings. The following table presents examples of practical recommendations to promote healthy daily exposure to natural light.

**Table:** Table of practical recommendations.


Encourage a morning outdoor routine	Spend 20-30 min outdoors within the first 1-2 h after waking. Morning light is particularly effective for reinforcing the body’s circadian clock.
Combine light exposure with existing habits	Taking a morning walk, drinking coffee on a balcony or patio, gardening, or walking a pet outdoors to make bright light exposure part of an established routine.
Promote active transportation	Walking or cycling to work, school, or nearby destinations instead of driving when practical and safe.
Encourage outdoor physical activity	Walking, jogging, hiking, biking, pickleball, or other forms of outdoor exercises provide the combined benefits of physical activity and natural light exposure.
Recommend a light therapy box when outdoor bright light is limited or impractical (eg, during winter, night-shift work, or poor weather).	Use a 10,000-lux therapeutic light box. Position the light approximately 30-60 cm (12-24 inches) from the face, according to the manufacturer’s instructions. Use the light shortly after waking for 20-30 min daily.

### Conclusion

The evidence linking natural light exposure and health presented in this article supports its consideration as a potential seventh pillar of lifestyle medicine, alongside healthy nutrition, physical activity, restoratives sleep, stress management, positive social relationships, and avoidance of harmful substances. The integration of natural light exposure into lifestyle medicine appears relevant given its direct influence on physiological and mental health and its compatibility with the other pillars. Indeed, the synthesis of the evidence presented in the article suggests that, much like other pillars of lifestyle medicine, exposure to natural light constitutes a modifiable lifestyle factor whose effects extend across multiple dimensions of optimal physiological functioning. It contributes to the regulation of key biological systems involved in sleep, metabolism, and cognition, and has been associated with disease prevention as well as mood and stress regulation. In this context, natural light exposure represents an accessible and feasible strategy that can be incorporated into daily activities, enabling both clinicians and patients to translate lifestyle medicine recommendations into practice. Based on current knowledge linking exposure to natural light and health, clinicians are already in a position to inform and guide patients toward strategies that promote more adequate light exposure, primarily through increased outdoor activities that combine sunlight exposure, exposure to open air, and physical exercise. Further research is required to better characterize the effects of natural light exposure on physical and mental health and the overall evidence presented in this article supports continued investigation in this field. To better understand the variability of light exposure effects according to environmental context, individual characteristics, and lifestyle habits, future studies should extend beyond laboratory settings and be conducted in real-world conditions. The emergence of wearable sensing technologies capable of continuously measuring parameters of natural and artificial light exposure opens new perspectives for capturing the complex interplay of factors that influence its effects on health and well-being.

## References

[1] Balabanoff D . Light and Embodied Experience in the Reimagined Birth Environment. University College Dublin; 2017. Thesis. https://www.researchgate.net/publication/327546110_Light_and_Embodied_Experience_in_the_Reimagined_Birth_Environment

[2] Blume C Garbazza C Spitschan M . Effects of light on human circadian rhythms, sleep and mood. Somnologie (Berl). 2019;23(3):147-156. doi:10.1007/s11818-019-00215-x. 31534436 PMC6751071

[3] Beute F de Kort YA . Salutogenic effects of the environment: review of health protective effects of nature and daylight. Applied Psychology Health and well-being. 2014;6(1):67-95. doi:10.1111/aphw.12019. 24259414

[4] Bertani DE De Novellis AMP Farina R , et al. Shedding light on light: a review on the effects on mental health of exposure to optical radiation. Int J Environ Res Publ Health. 2021;18(4):1670. doi:10.3390/ijerph18041670. PMC7916252 33572423

[5] Sundermann M Chielli D Spell S . Nature as medicine: the 7th (unofficial) pillar of lifestyle medicine. Am J Lifestyle Med. 2023;17(5):717-729. doi:10.1177/15598276231174863. 37711353 PMC10498981

[6] Schibler U . The daily rhythms of genes, cells and organs. Biological clocks and circadian timing in cells. EMBO Rep. 2005;6(Suppl 1):S9–S13. doi:10.1038/sj.embor.7400424. 15995671 PMC1369272

[7] Vandewalle G Maquet P Dijk DJ . Light as a modulator of cognitive brain function. Trends Cognit Sci. Trends in cognitive sciences. 2009;13(10):429-438. doi:10.1016/j.tics.2009.07.004. 19748817

[8] aan het Rot M Benkelfat C Boivin DB Young SN . Bright light exposure during acute tryptophan depletion prevents a lowering of mood in mildly seasonal women. Eur Neuropsychopharmacol. European neuropsychopharmacology. 2008;18(1):14-23. doi:10.1016/j.euroneuro.2007.05.003. 17582745

[9] Rüger M Scheer FA . Effects of circadian disruption on the cardiometabolic system. Rev Endocr Metab Disord. Reviews in endocrine & metabolic disorders. 2009;10(4):245-260. doi:10.1007/s11154-009-9122-8. PMC3026852 19784781

[10] Liu W Higashikuni Y Sata M . Chronobiological rhythms and artificial light at night in vascular physiology and pathology. Hypertens Res. Hypertension research. 2025;48(3):1204-1207. doi:10.1038/s41440-024-02040-8. 39633002

[11] Hubalek S Brink M Schierz C . Office workers’ daily exposure to light and its influence on sleep quality and mood. Light Res Technol. 2010;42(1):33-50. doi:10.1177/1477153509355632.

[12] Riemersma-van der Lek RF Swaab DF Twisk J Hol EM Hoogendijk WJ Van Someren EJ . Effect of bright light and melatonin on cognitive and noncognitive function in elderly residents of group care facilities: a randomized controlled trial. JAMA. 2008;299(22):2642-2655. doi:10.1001/jama.299.22.2642. 18544724

[13] Thorne HC Jones KH Peters SP Archer SN Dijk DJ . Daily and seasonal variation in the spectral composition of light exposure in humans. Chronobiol Int. 2009;26(5):854-866. doi:10.1080/07420520903044315. 19637047

[14] de Menezes-Júnior LAA Sabião TDS Carraro JCC Machado-Coelho GLL Meireles AL . The role of sunlight in sleep regulation: analysis of morning, evening and late exposure. BMC Public Health. 2025;25(1):3362. doi:10.1186/s12889-025-24618-8. 41053799 PMC12502225

[15] Chang AM Scheer FA Czeisler CA . The human circadian system adapts to prior photic history. J Physiol. 2011;589(Pt 5):1095-1102. doi:10.1113/jphysiol.2010.201194. 21224217 PMC3060589

[16] Schlangen LJM Price LLA . The lighting environment, its metrology, and non-visual responses. Front Neurol. 2021;12:624861. doi:10.3389/fneur.2021.624861. 33746879 PMC7970181

[17] Turner PL Mainster MA . Circadian photoreception: ageing and the eye's important role in systemic health. Br J Ophthalmol. 2008;92(11):1439-1444. doi:10.1136/bjo.2008.141747. 18757473 PMC2582340

[18] Vidafar P McGlashan EM Burns AC , et al. Greater sensitivity of the circadian system of women to bright light, but not dim-to-moderate light. J Pineal Res. 2024;76(2):e12936. doi:10.1111/jpi.12936. 39041348 PMC10909465

[19] Terzić Z Mikić M Ljaljević A Bojić MĐ Bojić M . The long-term efficacy of heliotherapy in ameliorating disease severity and improving the quality of life in patients with atopic dermatitis. Postȩpy Dermatol Alergol. 2023;40(1):159-164. doi:10.5114/ada.2022.124681. 36909908 PMC9993203

[20] Baggerly CA Cuomo RE French CB , et al. Sunlight and vitamin D: necessary for public health. J Am Coll Nutr. 2015;34(4):359-365. doi:10.1080/07315724.2015.1039866. 26098394 PMC4536937

[21] Kauffman JM . Benefits of vitamin D supplementation. J Am Phys Surg. Journal of American Physicians and Surgeons. 2009;14:38-45. https://www.grc.com/health/pdf/benefits_of_vitamin_d_supplementation.pdf

[22] Bustamante M Hernandez-Ferrer C Tewari A , et al. Dose and time effects of solar-simulated ultraviolet radiation on the in vivo human skin transcriptome. Br J Dermatol. 2020;182(6):1458-1468. doi:10.1111/bjd.18527. 31529490 PMC7318624

[23] Hoel DG Berwick M de Gruijl FR Holick MF . The risks and benefits of sun exposure 2016. Derm Endocrinol. 2016;8(1):e1248325. doi:10.1080/19381980.2016.1248325. PMC5129901 27942349

[24] Lindqvist PG Epstein E Nielsen K Landin-Olsson M Ingvar C Olsson H . Avoidance of sun exposure as a risk factor for major causes of death: a competing risk analysis of the Melanoma in Southern Sweden cohort. J Intern Med. 2016;280(4):375-387. doi:10.1111/joim.12496. 26992108

[25] Grant W Bhattoa H Boucher B . Seasonal variations of U.S. mortality rates: roles of solar ultraviolet-B doses, vitamin D, gene expression, and infections. J Steroid Biochem Mol Biol. 2017;173(2017):5-12. ISSN 0960-0760. doi:10.1016/j.jsbmb.2017.01.003. 28088363

[26] Scragg R Rahman J Thornley S . Association of sun and UV exposure with blood pressure and cardiovascular disease: a systematic review. J Steroid Biochem Mol Biol. 2019;187:68-75. doi:10.1016/j.jsbmb.2018.11.002. 30412763

[27] Cannistraci CV Nieminen T Nishi M , et al. Summer shift: a potential effect of sunshine on the time onset of ST-Elevation acute myocardial infarction. J Am Heart Assoc. 2018;7(8):006878. doi:10.1161/JAHA.117.006878. PMC6015398 29626152

[28] Freedman DM Dosemeci M McGlynn K . Sunlight and mortality from breast, ovarian, colon, prostate, and non-melanoma skin cancer: a composite death certificate based case-control study. Occup Environ Med. 2002;59(4):257-262. doi:10.1136/oem.59.4.257. 11934953 PMC1740270

[29] Wallis DW Penckofer S Sizemore GW . The “sunshine deficit” and cardiovascular disease. Circulation. 2008;118:1476-1485. 18824654 10.1161/CIRCULATIONAHA.107.713339

[30] Geldenhuys S Hart PH Endersby R , et al. Ultraviolet radiation suppresses obesity and symptoms of metabolic syndrome independently of vitamin D in mice fed a high-fat diet. Diabetes. 2014;63(11):3759-3769. doi:10.2337/db13-1675. 25342734

[31] Vanlint S . Vitamin D and obesity. Nutrients. 2013;5(3):949-956. doi:10.3390/nu5030949. 23519290 PMC3705328

[32] Afzal S Bojesen SE Nordestgaard BG . Low 25-hydroxyvitamin D and risk of type 2 diabetes: a prospective cohort study and metaanalysis. Clin Chem. 2013;59(2):381-391. doi:10.1373/clinchem.2012.193003. 23232064

[33] Caron-Jobin M Morisset A-S Tremblay A Huot C Légaré D Tchernof A . Elevated serum 25(OH)D concentrations, vitamin D, and calcium intakes are associated with reduced adipocyte size in women. Obesity. 2011;19:1335-1341. doi:10.1038/oby.2011.90. 21527900

[34] Arns M van der Heijden KB Arnold LE Kenemans JL . Geographic variation in the prevalence of attention-deficit/hyperactivity disorder: the sunny perspective. Biol Psychiatry. 2013;74(8):585-590. doi:10.1016/j.biopsych.2013.02.010. 23523340

[35] Hanford N Figueiro M . Light therapy and Alzheimer's disease and related dementia: past, present, and future. J Alzheimers Dis. 2013;33(4):913-922. doi:10.3233/JAD-2012-121645. 23099814 PMC3553247

[36] Keeney JTR Förster S Sultana R , et al. Dietary vitamin D deficiency in rats from middle to old age leads to elevated tyrosine nitration and proteomics changes in levels of key proteins in brain: implications for low vitamin D-dependent age-related cognitive decline. Free Radic Biol Med. 2013;65:324-334. doi:10.1016/j.freeradbiomed.2013.07.019. 23872023 PMC3859828

[37] Littlejohns TJ Henley WE Lang IA , et al. Vitamin D and the risk of dementia and Alzheimer disease. Neurology. 2014;83(10):920-928. doi:10.1212/WNL.0000000000000755. 25098535 PMC4153851

[38] Balion C Griffith LE Strifler L , et al. Vitamin D, cognition, and dementia: a systematic review and meta-analysis. Neurology. 2012;79(13):1397-1405. doi:10.1212/WNL.0b013e31826c197f. 23008220 PMC3448747

[39] Rosenthal NE Sack DA Gillin JC , et al. Seasonal affective disorder. A description of the syndrome and preliminary findings with light therapy. Arch Gen Psychiatry. 1984;41(1):72-80. doi:10.1001/archpsyc.1984.01790120076010. 6581756

[40] Terman M Terman JS . Light therapy for seasonal and nonseasonal depression: efficacy, protocol, safety, and side effects. CNS Spectr. 2005;10(8):647-672. doi:10.1017/s1092852900019611. 16041296

[41] Wirz-Justice A Graw P Kräuchi K , et al. Natural' light treatment of seasonal affective disorder. J Affect Disord. 1996;37(2-3):109-120. doi:10.1016/0165-0327(95)00081-x 8731073

[42] An M Colarelli SM O'Brien K Boyajian ME . Why we need more nature at work: effects of natural elements and sunlight on employee mental health and work attitudes. PLoS One. 2016;11(5):e0155614. doi:10.1371/journal.pone.0155614. 27214041 PMC4877070

[43] Engel P Fagherazzi G Mesrine S Boutron-Ruault MC Clavel-Chapelon F . Joint effects of dietary vitamin D and sun exposure on breast cancer risk: results from the French E3N cohort. Cancer Epidemiol Biomarkers Prev. 2011;20(1):187-198. *Cancer epidemiology, biomarkers & prevention : a publication of the American Association for Cancer Research, cosponsored by the American Society of Preventive Oncology* . doi:10.1158/1055-9965.EPI-10-1039. 21127286

[44] Rudders SA Camargo CA Jr . Sunlight, vitamin D and food allergy. Curr Opin Allergy Clin Immunol. 2015;15(4):350-357. doi:10.1097/ACI.0000000000000177. 26110686

[45] Grant WB . A review of the role of solar ultraviolet-B irradiance and vitamin D in reducing risk of dental caries. Derm Endocrinol. 2011;3(3):193-198. doi:10.4161/derm.3.3.15841. PMC3219170 22110779

[46] Kaufman HW Niles JK Kroll MH Bi C Holick MF . SARS-CoV-2 positivity rates associated with circulating 25-hydroxyvitamin D levels. PLoS One. 2020;15(9):e0239252. doi:10.1371/journal.pone.0239252. 32941512 PMC7498100

[47] Orton SM Wald L Confavreux C , et al. Association of UV radiation with multiple sclerosis prevalence and sex ratio in France. Neurology. 2011;76(5):425-431. doi:10.1212/WNL.0b013e31820a0a9f. 21282589 PMC3034408

[48] Lambert GW Reid C Kaye DM Jennings GL Esler MD . Effect of sunlight and season on serotonin turnover in the brain. Lancet (London, England). 2002;360(9348):1840-1842. doi:10.1016/s0140-6736(02)11737-5. 12480364

[49] van der Rhee H de Vries E Coomans C van de Velde P Coebergh JW . Sunlight: for better or for worse? A review of positive and negative effects of sun exposure. Cancer Res Front. 2016;2:156-183. doi:10.17980/2016.156.

[50] Kift RC Webb AR . Globally estimated UVB exposure times required to maintain sufficiency in vitamin D levels. Nutrients. 2024;16(10):1489. doi:10.3390/nu16101489. 38794727 PMC11124381

[51] Young AR Narbutt J Harrison GI , et al. Optimal sunscreen use, during a sun holiday with a very high ultraviolet index, allows vitamin D synthesis without sunburn. Br J Dermatol. 2019;181(5):1052-1062. doi:10.1111/bjd.17888. 31069787 PMC6899952

[52] Alfredsson L Armstrong BK Butterfield DA , et al. Insufficient sun exposure has become a real public health problem. Int J Environ Res Publ Health. 2020;17(14):5014. doi:10.3390/ijerph17145014. PMC7400257 32668607

[53] Pruszyński J Cianciara D Włodarczyk-Pruszyńska I Górczak M Padzińska-Pruszyńska I . Indoor generation era.Risks and challenges. J Edu Health Sport. 2023;48(1):23-40. doi:10.12775/JEHS.2023.48.01.002.

[54] Reiter RJ Tan DX Korkmaz A , et al. Light at night, chronodisruption, melatonin suppression, and cancer risk: a review. Crit Rev Oncog. 2007;13(4):303-328. doi:10.1615/critrevoncog.v13.i4.30. 18540832

[55] Al-Naggar RA Anil SH . Artificial light at night and cancer: global study. Asian Pac J Cancer Prev APJCP. 2016;17(10):4661-4664. doi:10.22034/apjcp.2016.17.10.4661. 27892680 PMC5454613

[56] Blask DE Dauchy RT Dauchy EM , et al. Light exposure at night disrupts host/cancer circadian regulatory dynamics: impact on the warburg effect, lipid signaling and tumor growth prevention. PLoS One. 2014;9(8):e102776. doi:10.1371/journal.pone.0102776. 25099274 PMC4123875

[57] Davis S Mirick DK . Circadian disruption, shift work and the risk of cancer: a summary of the evidence and studies in Seattle. Cancer Causes Control. 2006;17(4):539-545. doi:10.1007/s10552-005-9010-9. 16596308

[58] Kantermann T Roenneberg T . Is light-at-night a health risk factor or a health risk predictor? Chronobiol Int. 2009;26(6):1069-1074. doi:10.3109/07420520903223984. 19731106

[59] Wojciechowska W Hahad O Daiber A Rajzer M . Night light pollution and cardiovascular disease. Kardiol Pol. 2025;83(7-8):801-807. doi:10.33963/v.phj.106586. 40464453

[60] Salomon I Sam S Rehman YU Hope IM . Artificial light exposure at night: a hidden risk factor for type 2 diabetes. Sleep Med. 2025;X(10):100146. doi:10.1016/j.sleepx.2025.100146. PMC12221600 40606287

[61] Chen M Zhao Y Lu Q , et al. Artificial light at night and risk of depression: a systematic review and meta-analysis. Environ Health Prev Med. 2024;29:73. doi:10.1265/ehpm.24-00257. 39721676 PMC11701095

[62] Gangwisch JE . Invited commentary: nighttime light exposure as a risk factor for obesity through disruption of circadian and circannual rhythms. Am J Epidemiol. 2014;180(3):251-253. 24875372 10.1093/aje/kwu119

[63] Windred DP Burns AC Rutter MK , et al. Light exposure at night and cardiovascular disease incidence. JAMA Netw Open. 2025;8(10):e2539031. doi:10.1001/jamanetworkopen.2025.39031. 41129148 PMC12550636

[64] Figueiro MG Pedler D . Cardiovascular disease and lifestyle choices: spotlight on circadian rhythms and sleep. Prog Cardiovasc Dis. 2023;77:70-77. doi:10.1016/j.pcad.2023.02.004. 36841493 PMC10225333

[65] Arshad D Joyia UM Fatima S , et al. The adverse impact of excessive smartphone screen-time on sleep quality among young adults: a prospective cohort. Sleep Sci. 2021;14(4):337-341. doi:10.5935/1984-0063.20200114. 35087630 PMC8776263

[66] Rångtell FH Ekstrand E Rapp L , et al. Two hours of evening reading on a self-luminous tablet vs. reading a physical book does not alter sleep after daytime bright light exposure. Sleep Med. 2016;23:111-118. doi:10.1016/j.sleep.2016.06.016. 27539026

[67] Hébert M Martin SK Lee C Eastman CI . The effects of prior light history on the suppression of melatonin by light in humans. J Pineal Res. 2002;33(4):198-203. doi:10.1034/j.1600-079x.2002.01885.x. 12390501 PMC3925650

[68] Wickwire EM Geiger-Brown J Scharf SM Drake CL . Shift work and shift work sleep disorder: clinical and organizational perspectives. Chest. 2017;151(5):1156-1172. doi:10.1016/j.chest.2016.12.007. 28012806 PMC6859247

[69] Cyr M Artenie DZ Al Bikaii A Lee V Raz A Olson JA . An evening light intervention reduces fatigue and errors during night shifts: a randomized controlled trial. Sleep Health. 2023;9(3):373-380. doi:10.1016/j.sleh.2023.02.004. 37080863

[70] Urrutia-Moldes A . Light behind bars: how light impacts mental health in prisons. Int J Prison Health. 2025;21(3):347-362. doi:10.1108/IJOPH-11-2024-0074. 40302149

[71] Boubekri M Cheung IN Reid KJ Wang CH Zee PC . Impact of windows and daylight exposure on overall health and sleep quality of office workers: a case-control pilot study. J Clin Sleep Med. 2014;10(6):603-611. doi:10.5664/jcsm.3780. 24932139 PMC4031400

[72] Walch JM Rabin BS Day R Williams JN Choi K Kang JD . The effect of sunlight on postoperative analgesic medication use: a prospective study of patients undergoing spinal surgery. Psychosom Med. 2005;67(1):156-163. doi:10.1097/01.psy.0000149258.42508.70. 15673638

[73] Lech JC Halma MTJ Obajuluwa AO Baker M Hamblin MR . Fiat lux: light and pedagogy for the 21st century. Ann Neurosci. 2023;30(2):133-142. doi:10.1177/09727531221136646. 37706102 PMC10496794

[74] Żebrowska M Strohmaier S Westgarth C , et al. Timing and duration of dog walking and dog owner's chronotype in relation to incident depression risk among middle to older-aged female nurses. PLoS One. 2024;19(1):e0296922. doi:10.1371/journal.pone.0296922. 38295024 PMC10829988

